# Complex Interactions Between the Macrophyte *Acorus Calamus* and Microbial Fuel Cells During Pyrene and Benzo[*a*]Pyrene Degradation in Sediments

**DOI:** 10.1038/srep10709

**Published:** 2015-05-29

**Authors:** Zaisheng Yan, Helong Jiang, Haiyuan Cai, Yanli Zhou, Lee R. Krumholz

**Affiliations:** 1 State Key Laboratory of Lake Science and Environment, Nanjing Institute of Geography and Limnology, Chinese Academy of Sciences, Nanjing 210008, China; 2 Department of Microbiology and Plant Biology, University of Oklahoma, Norman, OK 73019, USA

## Abstract

This study investigated the interaction of the macrophyte *Acorus calamus* and sediment microbial fuel cells (SMFC) during the degradation of high molecular weight-polycyclic aromatic hydrocarbons (HMW-PAHs) in sediments. Over 367-days, the combination of macrophyte and SMFC led to an increase in pyrene and benzo[*a*]pyrene degradation rates by at least 70% compared to SMFC or macrophyte alone. While either the macrophyte or SMFC increased redox potential in sediments, redox potentials near the anode (approximately 6 cm depth) in the macrophyte-SMFC combination were markedly lower than that in the only macrophyte treatment. Moreover, rhizospheric bacterial communities in macrophyte-SMFC and macrophyte treatments were distinctly different. Aerobic genera (*Vogesella*, *Pseudomonas*, *Flavobacterium* and *Rhizobium)* and anaerobic genera (*Longilinea*, *Bellilinea*, *Desulfobacca* and *Anaeromyxobacter*) became dominant in the rhizosphere in macrophyte and macrophyte-SMFC treatments, respectively. In addition, the macrophyte-SMFC combination improved the numbers of not only aerobic but anaerobic PAHs degraders in sediments. So, the SMFC employment facilitated the formation of anoxic zones in sediments with oxygen loss and exudates from the roots. As a result, cooperation of anaerobic/aerobic microbial metabolism for accelerating HMW-PAHs removal occurred within sediments after combining macrophytes with SMFC.

Polycyclic aromatic hydrocarbons (PAHs) are ubiquitous organic pollutants that raise environmental concerns because of their toxicity, mutagenicity, and carcinogenicity^1^,^2^. In aquatic environments, PAHs generally accumulate in sediments due to their strong hydrophobicity and their resistance to biodegradation. High molecular weight (HMW) PAHs with more than four rings are particularly resistant to microbial degradation^3^,^4^. Recently, the concentrations of HMW-PAHs, such as pyrene and benzo[*a*]pyrene (B*a*P), in sediments of shallow lakes have shown an increasing trend^1^,^5^. Thus, removal of HMW-PAHs from contaminated sediments is becoming increasingly important to mitigate the associated risks.

To facilitate PAH removal from contaminated sediments/wetlands, phytoremediation and biostimulation techniques involving plants or electrodes have been proposed^6^,^7^,^8^. Previous studies have been successful using microbial anode of sediment microbial fuel cell (SMFC) to stimulate removal of hydrocarbons including diesel^9^,^10^, petroleum^11^, petroleum sludge^12^ or polycyclic aromatic hydrocarbons^13^. Mechanisms may involve either direct exoelectrogenic oxidation or syntrophic interactions within microbial consortia. A drawback of SMFC is the low flux of organic matter at the anode^14^. Moreover, the extent of SMFC assisted biodegradation of HMW-PAHs in sediments was limited to the dearth of readily degradable carbon compounds, which are known to stimulate HMW-PAHs degradation as co-substrates^3^,^13^.

Phytoremediation, is a cost-effective and eco-friendly remediation technology, and has been shown to have promise for elimination of organic pollutants from sediments^8^,^15^,^16^. Both rhizosphere oxygenation and secretion of root exudates have been shown to be important mechanisms during the stimulation of HMW-PAHs degradation in sediments^7^,^8^. Plant root exudates contain amino acids and other organic acids, some of which are known to stimulate bacterial population growth or facilitate co-metabolic degradation of HMW-PAHs^7^,^17^. In addition, root exudates can also enhance the desorption of PAHs from soils/sediments, increasing PAH biodegradability in the rhizosphere^18^,^19^.

Recently, it has been shown that plant root rhizodeposits or other exudates are used by a SMFC consisting of anodes in the rhizosphere and cathodes in the overlying oxic water^20^,^21^. In the plant SMFC, the plant root exudates are used as substrates by the electrochemically active bacteria associated with the anode^22^. Root associated rhizosphere oxygenation can also influence the activity of electrochemically active bacteria and cause changes in the anode potential^20^. These investigations studied the effects of plant growth on power production from SMFC, however little information is available on the interaction between macrophyte and SMFC during HMW-PAHs degradation in sediments.

The emergent macrophyte, sweet flag (*Acorus calamus*) is a wetland species that has been shown to have potential for phytoremediation of atrazine and purification of sewage due to its high level of biomass and well-developed root system^23^. In addition, sweet flag exhibits a high tolerance to petroleum hydrocarbons compounds and no oxidative damage to DNA was observed, even when stressed with high levels of diesel fuel^24^. Thus, the *A. calamus* may be useful for phytoremediation. However, little is known about its potential for the removal of HMW-PAHs from contaminated sediments, or its ability to produce root exudates.

In this study, the composition of root exudates of *A. calamus* and their effect on pyrene and B*a*P degradation was analyzed. Then, *A. calamus* was grown in a SMFC to investigate the interaction of macrophyte root and SMFC anode during pyrene and B*a*P degradation in freshwater sediments. In addition, the microbial community response to PAH and exposure to plant roots and SMFC was determined using 16S rRNA analysis.

## Results

### A. *calamus* growth and electricity production

The experiments lasted 367 days. The average root length, leaf length and total fresh weight of macrophytes at the end of experiments were shown in Supplementary Fig. S2. The total fresh weight of macrophytes in the macrophyte and macrophyte-SMFC treatments was 98.15 ± 21.06 g and 116.23 ± 15.09 g at the end of experiments, respectively, which were 2.1 and 2.5 times higher than those at the beginning of experiments. The root and leaf length in both the macrophyte and macrophyte-SMFC treatments at the end of experiments were 1.8-1.9 and 14.1-16.2 times higher than those at the beginning of experiments (Figure S2). Compared to the macrophyte treatment, the macrophyte-SMFC treatment was not significantly different in biomass, root or leaf length at the end of experiments.

The voltage produced in the SMFC and macrophyte-SMFC treatments were illustrated in Supplementary Fig. S3. Fluctuations in voltage output in both the SMFC and macrophyte-SMFC were observed throughout the experimental period. Voltages from macrophyte-SMFC were significantly higher than those from SMFC during the first 50 days (ANOVA, *p* = 0.000 < 0.05). However, after this period, slightly lower voltages from macrophyte-SMFC were observed. The average voltages from SMFC and macrophyte-SMFC over the entire period were 64.8 mV and 61.4 mV, respectively, and were not significantly different (ANOVA, *p* = 0.50).

### PAHs degradation kinetics

**Figure 1:**
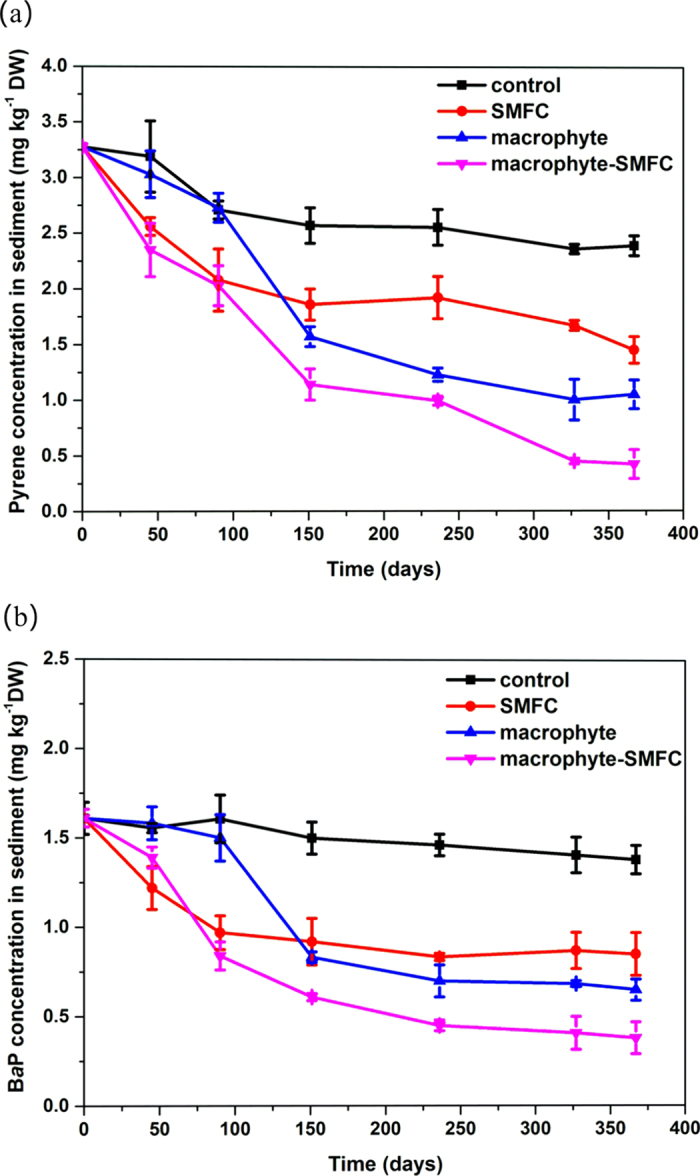
The concentrations of pyrene (**a**) and Bap (**b**) in sediments with different treatments over 367 days of experiments.

Pyrene and B*a*P content of sediments over the 367 days of the experiment are shown in Fig. 1. Although a slight decrease for pyrene was observed in the control treatment, the B*a*P contents did not decrease significantly (ANOVA, *p* = 0.48) during the 367-days. In comparison to the control treatment, the SMFC treatment led to the significant degradation of pyrene and B*a*P in sediments especially during the first 90 days. In the macrophyte treatment, the degradation of pyrene and B*a*P in sediments was similar to the control during the first 90 days, but significant degradation occurred after 150 days. At the end of experiments, pyrene and B*a*P removal efficiencies in the macrophyte-SMFC treatment reached 87.18 ± 5.62% and 76.40 ± 6.93% respectively, much higher than those in the control (Table 1). As shown in Table 1, pyrene and B*a*P removal efficiencies in the SMFC or macrophyte treatments were higher than those in the control but still lower than those in the macrophyte-SMFC treatment at the end of experiments.

Pyrene and B*a*P degradation kinetics were assessed by zero-order and first-order kinetic models fitted to the data in Fig. 1. The regression coefficients (*r*^2^) from first-order kinetic model ranged from 0.63 to 0.98 (Table 1) with those of the zero-order kinetic model varying from 0.54 to 0.91 (Table S3), suggesting that degradation followed first-order kinetics^25^. Compared to the control, the other three treatments led to markedly higher pyrene and B*a*P degradation rates. The macrophyte treatment resulted in significantly higher pyrene and B*a*P degradation rates than the SMFC treatment. Furthermore, the pyrene and B*a*P degradation rate in the macrophyte-SMFC treatment were at least 70% higher than those in the macrophyte treatment, and reached 0.00570 ± 0.00045 d^−1^ and 0.00514 ± 0.00073 d^−1^ respectively.

### Changes in ORP values in sediments

**Figure 2:**
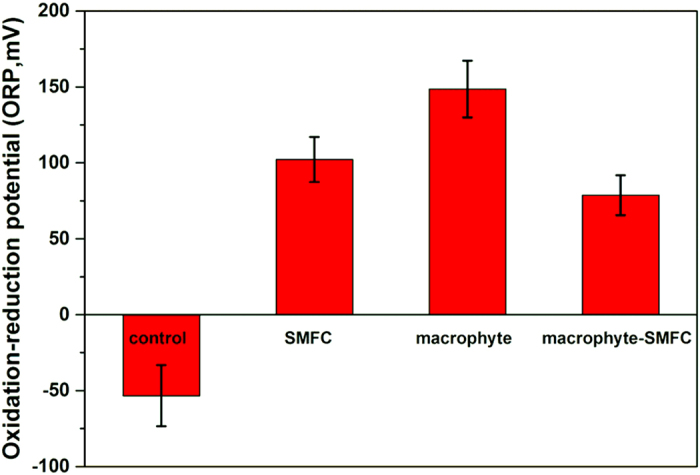
ORP in sediments measured at the end of experiment at approximately 6 cm depth.

Final time point ORP values in sediments from a depth of 6 cm are shown in Fig. 2. Anodes of SMFC were set at this sediment depth, so detected ORP values could reflect effects of the anode. The ORP value for the control was only –53 ± 20 mV and much less than those in the other three treatments. In comparison, the macrophyte treatment had the highest ORP value (149 ± 19 mV). While ORP values (approximate 79-102 mV) in the SMFC and macrophyte-SMFC treatments did not show substantial differences, the average ORP value in macrophyte-SMFC treatment was less than that in the SMFC treatment. For further confirmation, the other set of experiments was repeated as described in Supplementary Information, and it was still found that ORP values in the macrophyte-SMFC treatment were less than those in the macrophyte treatment (see Supplementary Fig. S4).

### Organic acid concentrations in pore water of sediments

As shown in Table 2, the concentrations of formate and acetate in pore water of sediments in the SMFC treatment were less than those in the control treatment on day 151, indicating anode-respiring microbes consumed the two acids. Acetate concentrations in the macrophyte treatment on day 151 reached a high of 38.25 ± 10.01 mg L^−1^, and then decreased to 14.12 ± 2.06 mg L^−1^ at the end of experiments. Concentrations of organic acids in the macrophyte-SMFC treatment were higher than those in the SMFC treatment but less than those in the macrophyte treatment. In addition, the residual concentrations of lactate for all treatments were not substantially different.

### PAH-degrading bacteria (PDB) numbers in sediments

**Figure 3:**
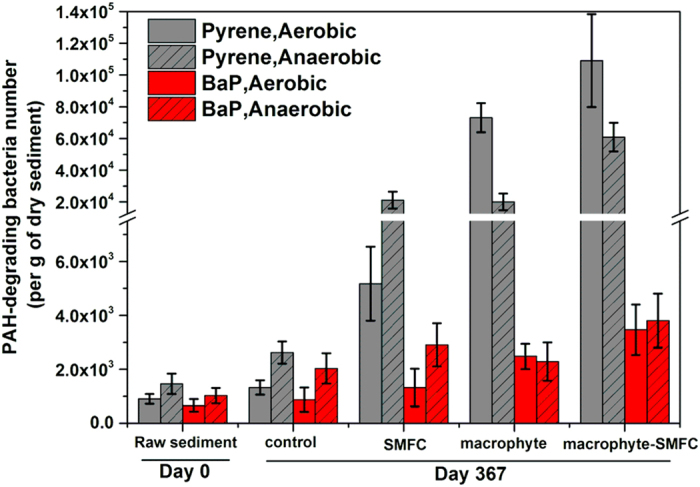
Bacterial population sizes in sediments under aerobic and anaerobic conditions.

The numbers of aerobic and anaerobic PDB was calculated with the MPN method for a bulk sediment sample on day 0 and for all sediment samples on day 367 (Fig. 3). The numbers of pyrene and B*a*P-degrading aerobic or anaerobic bacteria at the beginning and end of the experiment was similar in the control treatment. However, the numbers of pyrene-degrading aerobic bacteria in the other three treatments increased more than one order of magnitude. Pyrene-degrading aerobic bacteria in both macrophyte and macrophyte-SMFC treatments were at (7.3 ± 0.9) × 10^4^ and (10.9 ± 2.9) × 10^4^ cells per g of dry sediments respectively, and much higher than those in the SMFC treatment. Meanwhile, the aerobic B*a*P degraders also showed a substantial increase in the macrophyte and macrophyte-SMFC treatments but only a slight increase in the SMFC treatment.

Numbers of anaerobic pyrene and B*a*P-degrading bacteria increased significantly in all four treatments to varying degrees. Anaerobic pyrene degraders in the SMFC and macrophyte treatments were similar and approximately one order of magnitude higher than that in the control, but significantly lower than in the macrophyte-SMFC microcosms.

### Bacterial communities

After 367 days, bulk sediments, rhizosphere sediments and anode biofilms were used for microbial community analysis. A total of 329047 bacterial 16S rRNA gene sequences were obtained from the nine samples (see Supplementary Table S1). These sequences were assigned to 76431 OTUs with a cutoff of 0.03. Although none of the rarefaction curves reached a plateau (see Supplementary Fig. S5), Good’s Coverage estimators indicated that the sizes of libraries were sufficient to cover 84−90% of the bacterial communities (Table S1). Based on the Shannon diversity indices, diversity of sediment microbial communities increased with time. Rhizosphere sediments in the macrophyte-SMFC had the highest biodiversity among all samples (Table S1).

**Figure 4:**
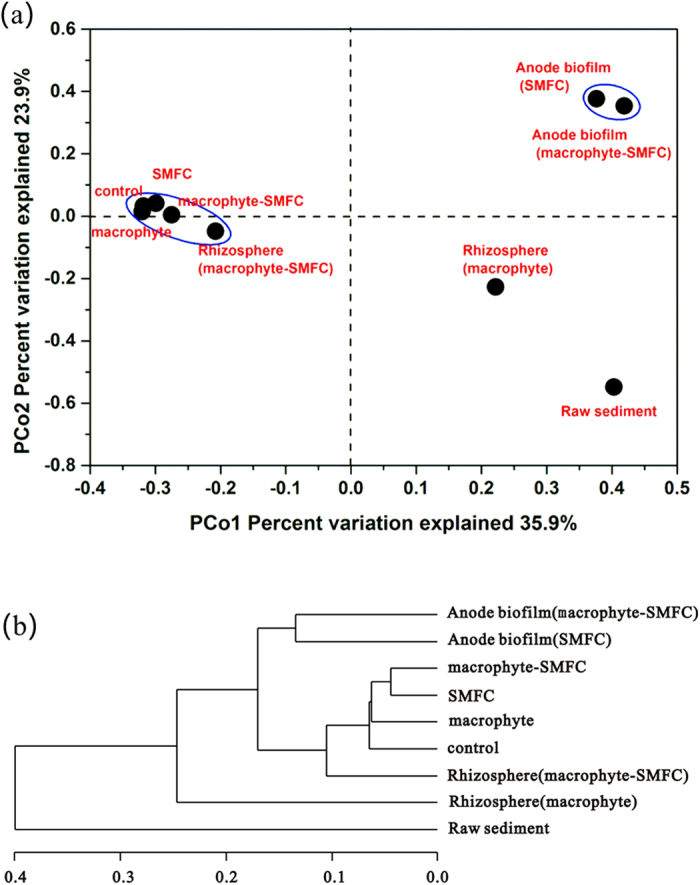
Principal coordinate analysis (PCoA) (**a**) and clusters of the bacterial communities of sediment samples and anode biofilms (**b**).

PCoA was used to compare the bacterial communities (Fig. 4). Nine different communities were grouped into four distinct clusters: (a) bulk sediments from the four treatments and rhizosphere sediments from the macrophyte-SMFC treatment; (b) anode biofilms from both SMFC and macrophyte-SMFC; (c) rhizosphere sediments from the macrophyte treatment; (d) raw sediments. Surprisingly, rhizosphere communities in the macrophyte-SMFC and macrophyte treatments did not cluster together.

**Figure 5:**
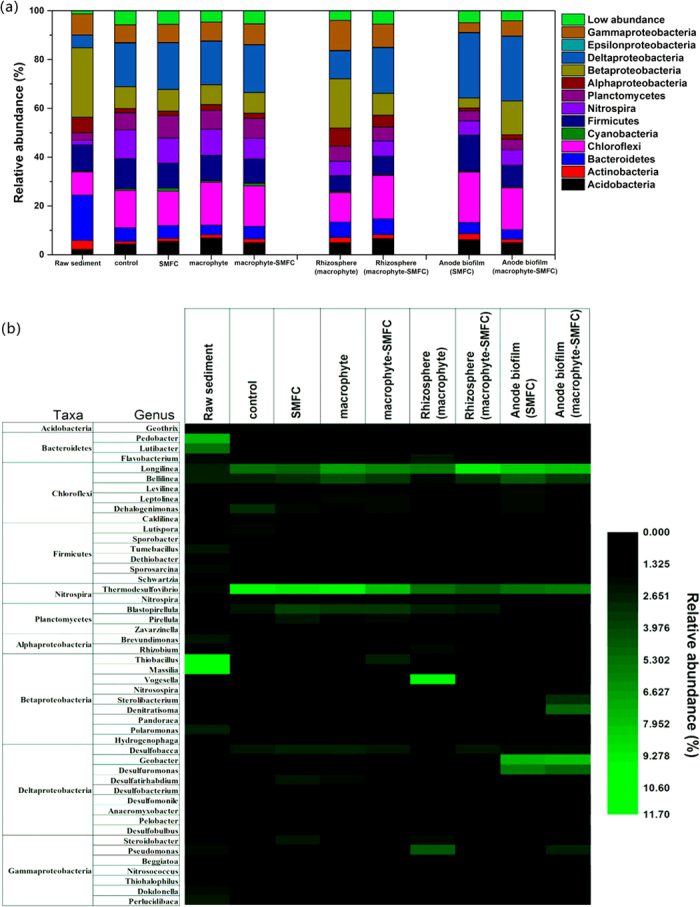
Relative abundance of bacterial phylogenetic groups at the phylum level and proteobacterial classes of sediment samples and anode biofilms (**a**) and the heat map at the genus level (**b**). Dominant groups are labeled, all minor components are clustered into “Low abundance”. The genera that are less than 1% of total composition in all libraries were not included in the heat map.

From the phylogenetic analysis, thirty-five bacterial phyla were detected in the nine libraries (Fig. 5a). The majority of sequences belonged to the *Proteobacteria* and accounted for 36.0-51.6% of the total reads in each bacterial community. *Betaproteobacteria* (20.1%) and *Deltaproteobacteria* (18.7%) were dominant classes in the rhizosphere of the macrophyte and macrophyte-SMFC treatments, respectively. *Deltaproteobacteria* (26.5%) were dominant on the anode biofilms.

The genus level characterization further illustrates variations in the bacterial community. A heatmap (Fig. 5b) shows the relative changes in abundance of bacterial genera among the samples. At the end of experiments, *Thermodesulfovibrio*, *Longilinea*, *Bellilinea* and *Desulfobacca* were dominant genera in bulk sediments. *Desulfobulbus* was more abundant in bulk sediments of the macrophyte-SMFC relative to other bacterial communities. *Vogesella* (10.34%), *Pseudomonas*, *Flavobacterium*, and *Rhizobium* were dominant genera in the rhizosphere sediments in the macrophyte rhizosphere, wheras *Longilinea* (9.42%), *Bellilinea*, *Desulfobacca*, and *Anaeromyxobacter* were dominant in the rhizosphere of the macrophyte-SMFC treatment. *Geobacter*, *Desulfuromonas*, *Longilinea*, and *Bellilinea* dominated the anode biofilms with *Denitratisoma* (4.8%) observed on the anode biofilm in the macrophyte-SMFC treatment.

### Characterization of *A. Calamus* root exudates and their effects on PAHs biodegradation

To determine the role of root exudates on PAH degradation, amino acids and fatty acids of root exudates from hydroponically grown *A. calamus* with and without pyrene and B*a*P exposure were identified (Table 3). Glycine (8.266 ± 0.194 μM L^−1^) and aspartic acid (3.837 ± 0.686 μM L^−1^) were the most abundant amino acids, while histidine, threonine, tyrosine were not detected in hydroponic systems without PAH exposure. A substantial increase in amino acid concentration was observed after PAH exposure. The main fatty acids present in root exudates were formate, acetate, citrate, and malate. The acetate concentrations were markedly higher in hydroponic systems after PAHs exposure.

The effect of root exudates on biodegradation of pyrene and B*a*P was determined (Table S2). After 15 days of incubation, biodegradation was increased with root exudates. Furthermore, PAHs removal was increased when bacteria were exposed to root exudates from PAH-exposed plants.

## Discussion

This study indicated that a combination of macrophyte-SMFC further increased the removal of pyrene and B*a*P in sediments relative to SMFC employment or *A. calamus* alone. The enhanced degradation of PAHs in sediments with the SMFC has been mainly attributed to the anode acting as a long-term electron acceptor, allowing PAHs to be more effectively oxidized within sediments^6^,^10^,^13^. On the other hand, degradation during the macrophyte treatment was strongly associated with plant developmental stages^15^,^26^. The qualitative and quantitative composition of root exudates is determined by the stage of plant development, physiological status and environmental factors^18^,^27^. In general, the root exudation rates are lowest at seedling stage and increase until flowering finally decreasing at maturity^28^. It seems likely that pyrene and B*a*P removal in sediments within the macrophyte treatments (Fig. 1) is associated with variations in levels of root exudates during different growth stages.

The observed changes in levels of root exudates (Table 3) were assumed to occur as a result of adaptive changes in response to pollutant stress^26^,^29^. Here, *A. calamus* root exudates stimulated pyrene and B*a*P degradation (Table S2). The relative amount of biostimulation appeared to be determined by the chemical composition of the root exudates (Table 3). The root exudates include secondary plant metabolites such as aromatic organic acids^27^,^30^, some of which are intermediates during the microbial metabolism of PAHs^4^,^27^. Their production can act to stimulate PAH biodegradation by inducing the expression of PAH degrading enzymes or by acting as co-metabolic substrates^17^,^30^.

Plant roots also release oxygen to sediments and this may have also contributed to the higher numbers of PDB capable of aerobic biodegradation of HMW-PAHs in sediments (Fig. 3) and the higher ORPs in the sediment layer (Fig. 2). The SMFC may also increase the redox potential near the anode (Figure S4), although the influence of the anode was likely over a smaller area than the plant roots. Sediment redox potential in the macrophyte-SMFC treatment has previously been observed to be influenced by both macrophyte rhizosphere oxygenation and anodic redox potential^20^. The fact that ORP values in sediments in macrophyte-SMFC treatment was markedly less than those in the macrophyte treatment, suggested that interactions between anode of SMFC and macrophyte rhizosphere were not synergism but complex. Actually, the complex interaction was also reflected especially from the rhizospheric microbial community structure.

Some important genera (*Pseudomonas*, *Flavobacterium,* and *Rhizobium*) in rhizosphere sediments during the macrophyte treatment are aerobic or facultatively bacteria. Some species of *Pseudomonas* and *Flavobacterium* are known to degrade fluoranthene, pyrene, and B*a*P when supplemented with other forms of organic carbon^3^,^4^. Additionally, one strain related to *Rhizobium meliloti* can also utilizes B*a*P and other aromatic compounds^31^. Amino acids are powerful chemoattractants for *Rhizobium*, potentially promoting bioaccessibility of PAHs in the rhizosphere^27^. The presence of the aerobic bacteria suggests that the plant roots support a community of aerobic PAH degrading bacteria. However, the dominant genera *Longilinea*, *Bellilinea*, *Desulfobacca* and *Anaeromyxobacter* in the rhizosphere sediments in the macrophyte-SMFC were obligate anaerobic bacteria, suggesting that the anode of SMFC may decrease macrophyte rhizosphere oxygenation. Although the roots penetrated the graphite felt anodes (Figure S1,b), oxygen release by roots did not markedly affect current generation in the macrophyte-SMFC after the initial 50 days of the experiment (Figure S3). Cluster analysis (Fig. 4b) showed big differences between the macrophyte only and the macrophyte-SMFC rhizosphere community, confirming an effect of the anodes on rhizosphere microbial community structure.

Distinctive microbial communities were selectively enriched on the anode biofilm (Fig. 5), indicating the likely presence of unique metabolic pathways at the anodes. An exoelectrogenic anode microbial community has been previously shown to use fatty acids through an electrode-reduction pathway^32^ leading to low pore water acetate concentrations, similarly observed here (Table 2). Facultative denitrifying bacteria such as *Denitratisoma* seen in the anode biofilm in the macrophyte-SMFC treatment can use O_2_ if it is available, which protected O_2_ sensitive exoelectrogenic bacteria like *Geobacter* against oxygen diffusion into anode biofilm and at the same time degraded sediment organic compounds. The genera *Longilinea* and *Bellilinea* (belonging to *Anaerolineae* class), dominated the rhizosphere sediments and the anode biofilm in the macrophyte-SMFC treatment. These genera are capable of degrading plant rhizodeposits (carbohydrates and amino acids)^33^, potentially increasing oxygen consumption and expanding the anaerobic region near the anode.

It is also likely that the combination of macrophyte-SMFC and PAH enriched for species that were capable of HMW-PAHs degradation. The dominant phyla *Proteobacteria*, *Chloroflexi*, *Firmicutes*, *Nitrospira* and *Bacteroidetes* (Fig. 5a) have been described as major bacterial degraders of HMW-PAHs in sediments^3^,^4^. The dominant genera *Thermodesulfovibrio*, *Longilinea*, *Belliline* and *Desulfobacca* (Fig. 5b) found in the samples are associated with anaerobic degradation of PAHs and oxidation of organic compounds^34^,^35^. *Geobacter* and *Desulfuromonas* enriched at the anodes are also important in the anaerobic degradation of aromatic hydrocarbons^6^.

SMFC have previously been shown to change the geochemical conditions in sediments^36^. For example, sulfate-reducing bacteria enriched on anodes have previously been linked to S^0^ oxidation with the electrode as electron acceptor and/or the ability to disproportionate S^0^ to sulfate and sulfide^37^. An increase in numbers of *Desulfobulbus* in sediments in the macrophyte-SMFC treatment (Fig. 5b) could potentially have led to a rise of sulfide concentrations in the rhizosphere. The sulfide could then have exerted stress on the roots, decreasing O_2_ production, or the sulfide could react with O_2_ (biotically or abiotically) directly reducing its level in the rhizosphere^36^,^38^.

In the rhizosphere, oxygen can also be rapidly used during the oxidation of readily available organic matter from rhizodeposition^21^. This accelerated oxygen consumption likely lead to the coexistence of healthy aerobic and anoxic zones in sediments with the macrophyte-SMFC treatment. The combination of anaerobic and aerobic treatments has been previously shown to be effective for recalcitrant contaminants removal^39^. The higher number of pyrene-degrading bacteria under aerobic and especially anaerobic conditions in the macrophyte-SMFC treatment indicated that an adequate combination of anaerobic/aerobic microbial metabolism for accelerating HMW-PAHs removal likely occurred within these macrophyte-SMFC containing sediments.

## Methods

### Sediment, water and macrophyte samples

Surface sediments (at 0–10 cm depth) and water samples were collected from Meiliang Bay (31°30′ N, 120°11′ E) in Taihu Lake (China), and transported to laboratory within several hours. After sieving at 2 mm, sediment samples were mixed. PAHs were added to the sediments, by mixing stock solutions 1000 mg L^−1^ of pyrene or B*a*P (98% purity, Alfa Acsar Co., UK) with acetonitrile, which was added drop-wise to wet sediments followed by mechanical mixing^40^ to final contents of pyrene and B*a*P reaching 4 and 2 mg kg^−1^ dry sediments respectively. Prior to experiments, the spiked sediment samples were stored in the dark for 40 days for partial aging.

*A. calamus* was also obtained from Taihu Lake, It was thoroughly washed to remove sediment particles attached to the plant and roots and then placed in vessels for indoor acclimation (310 Lux illumination, 12:12 h light/dark).

### Experimental design

Buckets with approximately 10 L volume were used as sediment microcosms to perform the pyrene and B*a*P degradation experiments. Four different treatments were used as illustrated in Supplementary Information Fig. S1A, including SMFC deployment as described below (SMFC treatment), *A. calamus* (macrophyte treatment), *A. calamus* and SMFC deployment (macrophyte-SMFC treatment), and no addition control to mimic the natural attenuation. Each microcosm contained 5000 g wet sediments amended with PAHs and 4 L surface water and treatments were done in triplicate. In the macrophyte and macrophyte-SMFC treatments, three plants of *A. calamus* with an average total fresh weight of 45 ± 2 g were introduced per microcosm. All microcosms were incubated (310 Lux illumination, 12:12 h light/dark) at ambient temperature.

SMFCs (Figure S1c) were installed as described in previous studies^13^,^41^. Briefly, a circular anode (a projection area of 380 cm^2^) and a circular cathode (265 cm^2^) were made of graphite felt (5 mm in thickness, Nengkang Carbon, Shanghai, China). Three round holes (7 cm, diameter) were cut and spaced evenly in the cathode in order to plant *A. calamus*. The anode was placed approximately 6 cm below the surface of sediments, while the cathode was approximately 1 cm above the sediment surface. In the macrophyte-SMFC treatment (Figure S1d), three *A. calamus* plants were planted in sediments through the holes in the cathode. These electrodes were connected via epoxy-encapsulated wires, and the voltage signal between the anode and cathode across an external load of 100 Ω was measured using a multimeter (model 2700, Keithley Instruments, Cleveland, OH, USA).

During experiments, sediment samples in each microcosm were taken at days 45, 90, 151, 236, 327 and 367. Dry weight (DW) of sediments was measured after drying samples in a vacuum freeze dryer (FD-1B-50, Boyikang, Beijing, China).

### Redox potential measurement in the sediment layer

At the end of the experiments, oxidation-reduction potentials (ORP) at approximately 6 cm depth of sediments during the illumination period were measured with platinum electrodes (FJA-4, Chuan-Di Instrument & Equipment CO., LTD, Nanjing, China) against Ag/AgCl reference electrode, and 2 min was allowed to reach equilibrium. Measured ORP were converted and reported versus a standard hydrogen electrode (SHE).

### Enumeration of PAH-degrading bacteria (PDB) number

Bacterial counts from triplicate bulk sediment samples were performed using a most probable number (MPN) method^40^, with details for aerobic and anaerobic cultivation provided in the Supplementary Information.

### *A. calamus* root exudate analysis

To characterize *A. calamus* root exudates, root exudates were first harvested using a hydroponic system^18^, with additional details provided in the Supplementary Information. After two months incubations, root exudates were collected from each flask and analyzed. The effect of root exudates on the biodegradation of pyrene and BaP by sediment bacteria was studied using this same root exudate solution with details given in Supplementary Information.

### Bacterial community structure analysis

At the end of experiments, bulk sediment samples, rhizosphere sediment samples and anode biofilm samples were collected from sediment microcosms. The anode electrodes were rinsed with sterile water to remove visible sediments on the surface, and then a sterile razor blade was used to scrape the electrodes vigorously to acquire a complex consisting of carbon felt and electrode-associated microbes for subsequent DNA extraction. Bulk sediments, rhizosphere sediments and anode biofilm samples were immediately stored at −80 ^°^C for subsequent DNA extraction.

Microbial DNA was extracted with a PowerSoil^®^DNA Isolation Kit (MO BIO) according to manufacturer’s protocols. DNA concentration was determined with a Nanodrop 2000 (NanoDrop Technologies, Wilmington, USA). The V4-V5 regions of bacterial 16S ribosomal RNA genes were PCR amplified (95 °C for 2 min, followed by 25 cycles at 95 °C for 30 s, 55 °C for 30 s, and 72 °C for 45 s and a final extension at 72 °C for 10 m) using primers 515F 5’-barcode- GTGCCAGCMGCCGCGG)-3’ and 907R 5’-CCGTCAATTCMTTTRAGTTT-3’, where barcode is an eight-base sequence unique to each sample. PCR reactions were performed in triplicate 20 μL mixtures and contained 4 μL of 5 × FastPfu Buffer, 2 μL of 2.5 mM dNTPs, 0.8 μL of each primer (5 μM), 0.4 μL of FastPfu Polymerase, and 10 ng of template DNA. Amplicons were extracted from 2% agarose gels and purified using the AxyPrep DNA Gel Extraction Kit (Axygen Biosciences, Union City, CA, U.S.) according to the manufacturer’s instructions and quantified using QuantiFluor™ -ST (Promega, U.S.). Purified amplicons were pooled in equimolar amounts and paired-end sequenced (2 × 250) on an Illumina MiSeq platform. The raw reads were deposited into the NCBI Sequence Read Archive (SRA) database (Accession Number: SRP046042). Protocols for sequence processing and bioinformatics analysis can be found in the Supplementary Information.

### Analytical methodology and statistical analysis

The biodegradation of PAHs was described by two kinetic models including zero-order and first-order kinetics equation^13^,^25^,^40^ and as described in the Supplementary Information. Pyrene and B*a*P in sediment samples were extracted as described previously^13^,^40^, with modifications in the Supplementary Information.

The amino acid content in the root exudates was analyzed by o-phthalaldehyde (OPA) pre-column derivatization and HPLC (Agilent 1100, USA) analysis. The HPLC used a 4.6 × 250 mm Hypersil ODS-2 (*C*_18_) column with gradient elution. Solvent A (25 mM phosphate buffer pH 7.2 with 0.75% tetrahydrofuran) and solvent B (methanol to acetonitrile to 25 mM phosphate buffer pH 7.2, 45:45:10,vol.%) were pumped at initial ratio of A:B at 93:7, v/v, at a flow rate of 1 mL min^−1^ at 35 °C with UV detection (338 nm). Detection limits were 0.0052~0.6250 μM mL^−1^. The exudates sample was concentrated by freeze drying and re-dissolved in 0.5 mL deionized water for analysis.

Concentration of low-molecular-weight organic acids in plant root exudates and pore water of sediments were quantified by HPLC (Agilent 1200, USA) fitted with a reverse phase C_18_ column (EclipseXDB-C_18_, 4.6 × 150 mm, 5 μm) using 0.2% H_3_PO_4_- acetonitrile (5:95, v/v) as the mobile phase at a flow rate of 1 mL min^−1^. The detection wavelength was 215 nm and the column temperature was 25 °C.

Statistical significance of differences was determined by a one-way ANOVA analysis of variance using the SPSS software (IBM SPSS Statistics 19). A *p* < 0.05 was considered significant.

## Additional Information

**How to cite this article**: Yan, Z. *et al*. Complex Interactions Between the Macrophyte *Acorus calamus* and Microbial Fuel Cells During Pyrene and benzo[*a*]Pyrene Degradation in Sediments. *Sci. Rep*. **5**, 10709; doi: 10.1038/srep10709 (2015).

## Supplementary Material

Supplementary Information

## Figures and Tables

**Table 1:** The first-order rate constant (*k*, day^−1^) of PAH-degradation, half-lives (*t*_1/2_, day), correlation coefficient (*r*^*2*^) and final removal.

Treatment	Pyrene				B*a*P			
	*k* (d^−1^)	*r*^2^	*t*_1/2_ (d)	Final removal (%)	*k* (d^−1^)	*r*^2^	*t*_1/2_ (d)	Final removal (%)
control	0.00091 ± 0.00018	0.81	761.7	27.03 ± 4.06	0.00042 ± 0.00005	0.91	1650.4	14.29 ± 9.64
SMFC	0.00200 ± 0.00043	0.80	346.6	55.73 ± 5.65	0.00176 ± 0.00053	0.63	393.8	47.20 ± 8.32
macrophyte	0.00300 ± 0.00050	0.93	231.0	67.94 ± 5.28	0.00306 ± 0.00057	0.86	226.5	59.63 ± 3.61
macrophyte-SMFC	0.00570 ± 0.00045	0.98	121.6	87.18 ± 5.62	0.00514 ± 0.00073	0.93	134.9	76.40 ± 6.93

Data are means ± standard deviation.

**Table 2:** The concentrations of formate, lactate, acetate, and propionate in pore water of sediments under various treatments.

Time	Treatment	Formate (mg L^−1^)	Lactate (mg L^−1^)	Acetate (mg L^−1^)	Propionate (mg L^−1^)
0 day					
	Raw sediment	4.64 ± 0.76	74.86 ± 12.51	33.19 ± 8.78	4.20 ± 0.34
151 day					
	control	3.56 ± 0.21	54.19 ± 9.38	20.21 ± 5.43	3.89 ± 0.56
	SMFC	0.43 ± 0.03	40.21 ± 10.21	10.03 ± 3.92	4.01 ± 0.03
	macrophyte	5.87 ± 1.24	43.67 ± 8.05	38.25 ± 10.01	3.56 ± 0.28
	macrophyte-SMFC	4.02 ± 0.52	32.84 ± 6.32	28.43 ± 8.43	2.59 ± 0.73
367 day					
	control	ND^a^	15.55 ± 6.21	10.36±4.02	ND
	SMFC	ND	19.31 ± 3.19	ND	ND
	macrophyte	ND	16.86 ± 4.02	14.12 ± 2.06	ND
	macrophyte-SMFC	ND	13.61 ± 6.03	7.49 ± 1.04	ND

Data are means ± standard deviation

^a^ND, not detected, below the detection limit

**Table 3:** Amino acids and fatty acids composition of root exudates from sweet flag (*A. calamus*) cultivated in hydroponic systems after 2 months.

Class of compounds	Single compounds	Concentration (μM L^−1^)	
		Without PAH addition	With PAH addition
Amino acids			
	Aspartic acid	3.837 ± 0.686	7.294 ± 1.270
	Glutamic acid	1.205 ± 0.433	2.997 ± 0.054
	Serine	0.986 ± 0.106	2.966 ± 0.932
	Histidine	ND^a^	0.453 ± 0.030
	Glycine	8.266 ± 0.194	9.590 ± 0.637
	Threonine	ND	0.742 ± 0.036
	Alanine	0.294 ± 0.035	1.907 ± 0.091
	Arginine	1.599 ± 0.329	1.842 ± 0.050
	Tyrosine	ND	0.345 ± 0.032
	Cysteine	0.518 ± 0.159	0.294 ± 0.001
	Valine	1.096 ± 0.583	5.228 ± 0.116
	Methionine	1.198 ± 0.106	1.949 ± 0.566
	Phenylalanine	0.254 ± 0.028	1.865 ± 0.067
	Isoleucine	0.208 ± 0.024	0.891 ± 0.042
	Leucine	0.270 ± 0.030	1.019 ± 0.188
	Lysine	0.388 ± 0.037	0.586 ± 0.056
Fatty acids			
	Formic acid	0.303 ± 0.013	0.270 ± 0.037
	Acetic acid	1.057 ± 0.094	3.049 ± 0.108
	Citric acid	0.103 ± 0.006	0.103 ± 0.023
	Malic acid	0.234 ± 0.011	0.433 ± 0.004

Data are means ± standard deviation

^a^ND, not detected, below the detection limit.
