# Supplementary material for: Complex Interactions Between the Macrophyte Acorus Calamus and Microbial Fuel Cells During Pyrene and Benzo[a]Pyrene Degradation in Sediments
Source: Sci Rep. 2015 May 29;5:10709. doi: 10.1038/srep10709 (PMC4448661; doi:10.1038/srep10709)
Supplement: Supplementary Information [file srep10709-s1.pdf]

Supplementary Information for Publication

Number of pages: 15
Number of figures: 5
Number of tables: 3

Complex interactions between the macrophyte *Acorus calamus* and microbial fuel cells during pyrene and benzo[*a*]pyrene degradation in sediments

Zaisheng Yan [1], Helong Jiang [1*], Haiyuan Cai [1], Yanli Zhou [1] & Lee R Krumholz [2]

[1]State Key Laboratory of Lake Science and Environment, Nanjing Institute of Geography and Limnology, Chinese Academy of Sciences, Nanjing 210008, China

[2]Department of Microbiology and Plant Biology, University of Oklahoma, Norman, OK 73019, USA

[*]Corresponding author. Mail address: Nanjing Institute of Geography and Limnology, Chinese Academy of Sciences, 73 East Beijing Road, Nanjing 210008, China. Tel./fax: 0086-25-8688 2208. E-mail: hljiang@niglas.ac.cn (H.L. Jiang).

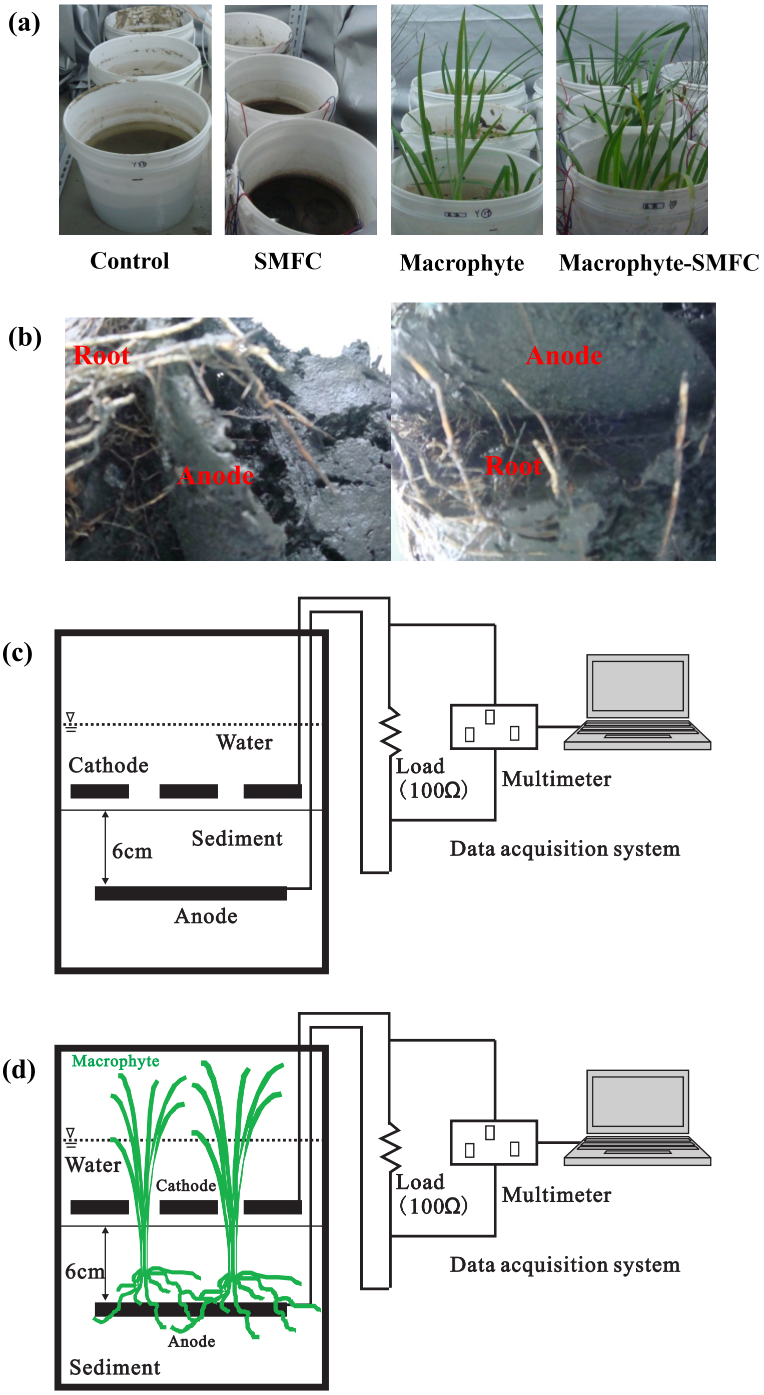

Figure S1 (a, b, c, d). Schematic of four different treatments in microcosms experiments (a), the backside of an anode graphite felt showing that *Acorus calamus* roots penetrated the anode (b), schematic diagram of the SMFC (c), and schematic diagram of the macrophyte-SMFC (d).

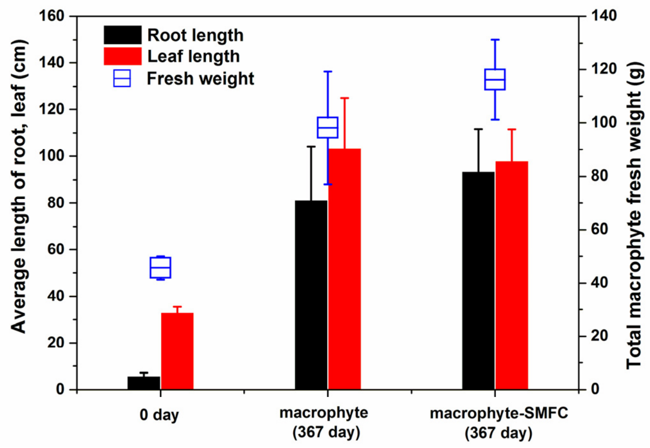

Figure S2. The average length of root and leaf, and total macrophyte fresh weight during the experiments.

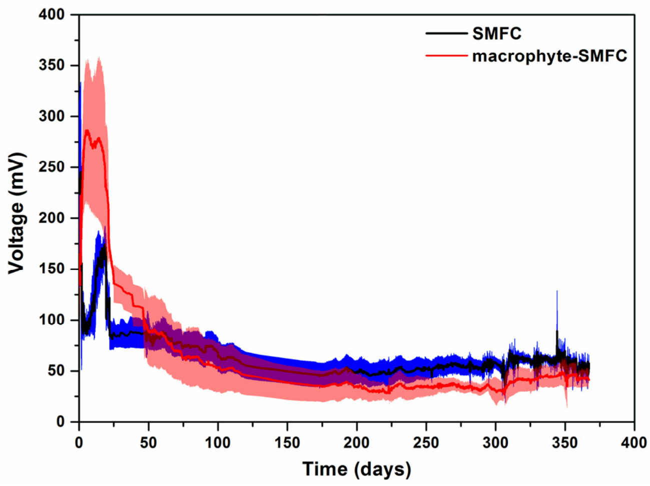

Figure S3. Voltage outputs of SMFC and macrophyte-SMFC during the 367 days operation. The fixed external resistance was 100 Ω.

Separate experiments were carried out in 1-litre beakers containing a sediment layer with 13 cm deep (Figure S4b) in order to further confirmation of oxidation-reduction potential (ORP) values in the macrophyte-SMFC and macrophyte treatments. The vertical profiles of ORP from the water-sediment interface to a depth of 6 cm with 1 cm intervals were acquired in four different treatments.

(a)

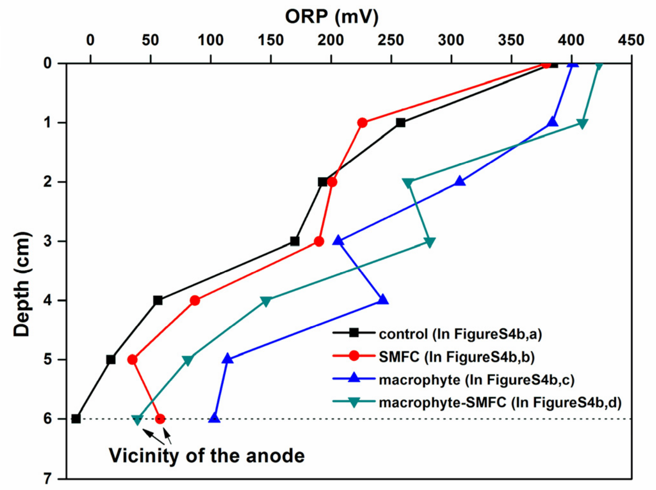

(b)

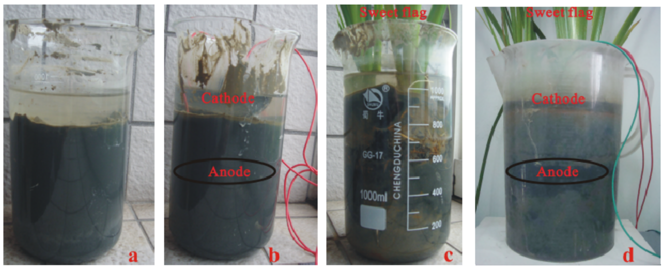

Figure S4 (a,b). The vertical profiles of ORP in sediments with four different treatments (a), and separate experiments in beakers for determining vertical profiles of ORP in sediments (b).

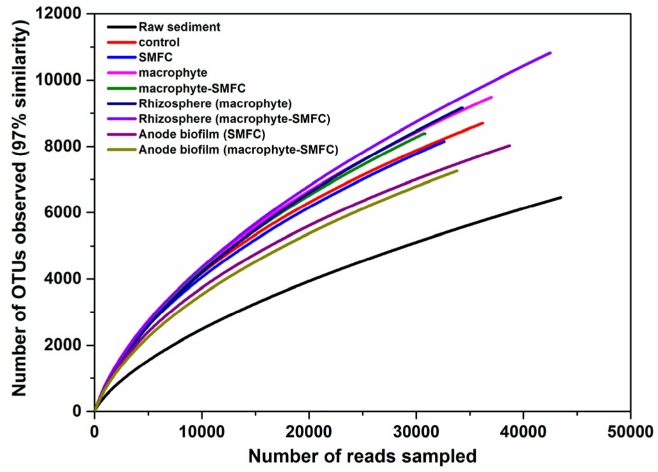

Figure S5. Rarefaction curves of the OTUs number at 97 % similarity for every group.

Table S1. Similarity-based OTUs and species richness and diversity estimates.

Sample	0.03 distance					
	Number of sequences	OTUs	ACE	Chao1	Shannon	Coverage
Raw sediment	43486	6457	26252	16164	6.51	0.9069
control	36162	8697	21559	15788	8.14	0.8739
SMFC	32559	8144	24091	16809	8.05	0.8589
macrophyte	36981	9489	27815	19327	8.21	0.8553
macrophyte-SMFC	30703	8391	26363	18473	8.11	0.8414
Rhizosphere(macrophyte)	34251	9165	28825	19374	7.81	0.8406
Rhizosphere(macrophyte-SMFC)	42460	10809	39623	25306	8.25	0.8455
Anode biofilm(SMFC)	38677	8023	20782	15090	7.77	0.8906
Anode biofilm(macrophyte-SMFC)	33768	7256	21409	14616	7.52	0.8794
Total	329047	76431	236719	160947		

Table S2. **Effects of *A. calamus* root exudates on pyrene and B*a*P biodegradation after 15-day aerobic incubation.**

PAHs	Initial concentrations (mg kg^{-1})	Without root exudates (mg kg^{-1})	Root exudates (mg kg^{-1})	
			non-PAHs-exposed	PAHs-exposed
Pyrene	2.39±0.09	2.06±0.03	1.65±0.13	1.05±0.17
B*a*P	1.38±0.08	1.29±0.06	1.01±0.08	0.78±0.02

Data are means ± standard deviation.

Table S3. **The zero-order rate constant (*k*, day^{-1}) of PAH-degradation, half-lives ($t_{1/2}$, day) and correlation coefficient (r^2).**

Treatment	Pyrene			BaP		
	k (d^{-1})	r^2	$t_{1/2}$ (d)	k (d^{-1})	r^2	$t_{1/2}$ (d)
control	0.00237±0.00050	0.78	292.5	0.00063±0.00008	0.91	1100.2
SMFC	0.0038±0.00093	0.72	182.4	0.00159±0.00055	0.54	435.9
macrophyte	0.00664±0.00105	0.87	104.4	0.00297±0.00059	0.80	233.4
macrophyte-SMFC	0.00719±0.00105	0.88	96.4	0.00318±0.00070	0.77	218.0

Data are means ± standard deviation.

Supplementary Methods

Enumeration of PAH-degrading bacteria (PDB) number. Mineral salt medium (MSM) agar plate was prepared as following: $(NH_4)_2SO_4$, 18 mM; $FeSO_4 \cdot 7H_2O$, 1 μM; $CaCl_2 \cdot 2H_2O$, 100 μM; $MgSO_4 \cdot 7H_2O$, 1 mM; $FeCl_3 \cdot 6H_2O$, 1 mM; NaCl, 0.85 mM; $MnCl_2 \cdot 4H_2O$, 7 μM and $Na_2MoO_4 \cdot 2H_2O$, 5 μM in 10 mM Na_2HPO_4-KH_2PO_4 buffer, pH 7.0. To detach microbial cells from sediments, fresh sediment sample with a 10 g of wet weight was added to 90 mL Bushnell-Haas Broth in sterile 200 mL screw-cap bottles, followed by shaking at 200 r min^{-1} for 15 min. Then a series of 10-fold dilutions (from 10^{-2} to 10^{-8} dilution) was prepared using sterile Bushnell-Haas Broth. Each MSM agar plate with 1.5% agar was divided into 20 sections by drawing four horizontal lines and three vertical lines on the back of the plate.

Mineral salt medium (MSM) agar plates were sprayed with acetonitrile containing pyrene (100 mg L^{-1}) or B*a*P (20 mg L^{-1}). The acetonitrile was evaporated immediately and a white spot was left on the plate. An aliquot of 10 μL diluted culture was dropped onto the spot, and each dilution had five replicates for MPN calculation. The spot was marked as positive if the colony was surrounded by a clear zone. The plates for growth of aerobic bacteria were incubated at 28 °C for 15 days. For anaerobic cultivation, the plates were placed inside an anaerobic jar and incubated at 28 °C in an anaerobic chamber for 38 days. The total number of positive growths in each dilution was counted and the bacterial population was calculated by a MPN program.

***A. calamus* root exudates analysis.** To examine changes in *A. calamus* root exudates due to PAH induced stress, one *A. calamus* plant with a fresh weight of 19.48 ± 2.65 g was transferred to a flask containing 300 mL sterile half-strength Hoagland's nutrient solution with addition of pyrene and B*a*P (each at a concentration of 1.0 mg L^{-1}) or without addition of PAHs and grown in an incubation chamber (25 °C, 800 lx, 12:12 h light/dark) for two months. Three identical hydroponic systems were prepared for each treatment. Prior to growing hydroponically, the root mass of each plant was cleaned of sediments by hand washing with sterile deionized water. Flasks were covered with aluminum foil to maintain dark conditions and prevent algal growth in the Hoagland's nutrient solution.

PAHs biodegradation experiments with root exudates. To determine the biodegradation of pyrene and B*a*P by indigenous bacteria in the presence or absence of *A. calamus* root exudates, 2 g of sediments (sedimentary sample from the control treatment on day 367) was suspended in 100 mL of sterile half-strength Hoagland's nutrient solution or *A. calamus* root exudates. The root exudates were used from PAHs-exposed plants or unexposed plants. The suspensions were placed in 150 mL Erlenmeyer flasks under sterile conditions, and each treatment was performed in duplicate. The flasks were sealed with Teflon-lined stoppers and were maintained at 120 r min^{-1} and 28 °C in the dark. At the end of the incubation period (15 days), the concentrations of pyrene and B*a*P present in the sediment mixture suspension were determined.

Sequencing data processing and bioinformatics analysis. Raw fastq files were demultiplexed, quality-filtered using QIIME (version 1.17) with the following criteria: (i) The 250 bp reads were truncated at any site receiving an average quality score <20 over a 10 bp sliding window, discarding the truncated reads that were shorter than 50 bp; (ii) exact barcode matching, 2 nucleotide mismatch in primer matching, reads containing ambiguous characters were removed; (iii) only sequences that overlap longer than 10 bp were assembled according to their overlap sequence. Reads which could not be assembled were discarded.

Operational Units (OTUs) were clustered with 97% similarity cutoff using MOTHUR version 1.31.2 [1] and chimeric sequences were identified and removed using UCHIME [2]. The phylogenetic affiliation of each 16S rRNA gene sequence was analyzed by RDP Classifier (http://rdp.cme.msu.edu/) against the SILVA 16S rRNA database [3]. On the basis of these clusters, rarefaction curves, OTUs, ACE, Chao1, Shannon diversity index and Good's coverage were generated using MOTHUR for each sample. Principal Coordinates Analysis (PCoA) was also conducted to depict patterns of beta diversity for bacterial communities. Cluster analysis in R statistical software (http://www.r-project.org/) with making the distance matrix using Bray-Curtis and applying the agglomerative clustering algorithm.

Analytical methodology and biodegradation kinetics of PAHs. Sediment samples were freeze dried and homogenized for analysis of PAHs in sediment. Two grams of sediment samples were transferred into glass centrifugal tube containing 10 mL of

acetonitrile, and the tube was placed in an ultrasonic bath with ultrasonication for 2 h. Following extraction, the tubes were subject to centrifugation for 20 min at 4000 r min^{-1}, and the supernatant were completely condensed to approximately 2 mL, followed by solid phase extraction column for clean-up, and concentrated, passed through a 0.22 μm nylon syringe filter and transferred to autosampler vials (1 mL) for analysis.

PAHs were analyzed with a high-performance liquid chromatograph (HPLC) (Agilent 1200, USA) fitted with a 4.6×150 mm reverse phase C_{18} column using 8:2 acetonitrile:water (v:v) as the mobile phase at a flow rate of 1 mL min^{-1} at 30 °C. The HPLC analysis was conducted with combination of UV and fluorescence detection. The detection wavelength was 254 nm for UV detector. For fluorescence detector, the excitation and emission wavelengths for pyrene were 260 nm and 380 nm, respectively. For B*a*P, the excitation and emission wavelengths were 290 nm and 410 nm, respectively. All data were subject to strict quality control procedures. The recoveries of the extraction method for pyrene were 90.3 ± 6.5 % from sediments, and 86.2 ± 2.3 % from water. For B*a*P, the recoveries of the extraction were 86.5 ± 7.3 % from sediments, and 82.8 ± 5.4 % from water. The detection limits were 0.1-5.0 μg kg^{-1} dry weight for sediments and 0.1 μg L^{-1} for water.

The biodegradation of PAHs was described by the zero order kinetics Eq. (1),

$$C = C_0 - kt \tag{1}$$

The biodegradation of PAHs was described by the first order kinetics Eq. (2),

$$C = C_0 e^{-kt} \tag{2}$$

where C_0 is the concentration of PAH at time zero, C is the concentration of PAH after subtracting the abiotic loss at time t, and k is the first-order rate constant (biodegradation rate) of the reaction. The half life for each PAH compound, $t_{1/2}$, was calculated by the following formula (3),

$$t_{1/2} = (\ln 2)/k \tag{3}$$

1 Schloss, P. D. *et al.* Introducing mothur: open-source, platform-independent, community-supported software for describing and comparing microbial communities. *Appl. Environ. Microbiol.* **75**, 7537-7541 (2009).

2 Edgar, R. C., Haas, B. J., Clemente, J. C., Quince, C. & Knight, R. UCHIME improves sensitivity and speed of chimera detection. *Bioinformatics* **27**, 2194-2200 (2011).

3 Amato, K. R. *et al.* Habitat degradation impacts black howler monkey (Alouatta pigra) gastrointestinal microbiomes. *ISME J.* **7**, 1344-1353 (2013).
